# Dr. Herman Delmar Hunt

**Published:** 1915-11

**Authors:** 


### OBITUARY

---

**Readers are requested to report promptly the death of all physicians in Western New York, or former residents of this region, or graduates of any medical school in Western New York, and to notify the families of the deceased of our desire to publish adequate obituary notices.**

---

**Dr. Herman Delmar Hunt,** Syracuse 1874, died at his home in Preble, N. Y., July 30, from angina pectoris, aged 68. He had been post master for 19 years.

